# ‘Physical activity, that’s a tricky subject.’ Experiences of health care professionals with physical activity in type 2 diabetes care

**DOI:** 10.1186/s12913-018-3102-1

**Published:** 2018-04-23

**Authors:** Mirjam Stuij

**Affiliations:** 1 Department of Medical Humanities, APH Research Institute, VU University medical centre, De Boelelaan 1089a, 1081 HV Amsterdam, the Netherlands; 2 Mulier Institute, Herculesplein 269, 3584 AA Utrecht, the Netherlands

**Keywords:** Experiences of healthcare professionals, Type 2 diabetes, Physical activity, Qualitative design, In-depth interviews, Narrative analysis

## Abstract

**Background:**

Based on a growing body of epidemiological and biomedical studies, physical activity (PA) is considered a cornerstone in type 2 diabetes treatment. However, it is also a practice embedded in daily life and, as such, may produce certain frictions as a topic in health care. The aim of this article is to give in-depth insight into experiences of health care professionals with the delivery of PA counselling to people with type 2 diabetes.

**Methods:**

This study is based on in-depth interviews with 24 Dutch professionals providing care to people with type 2 diabetes. They were asked to tell about their experiences with PA in different roles, both in their professional and personal lives. The interviews were audio-recorded and transcribed verbatim. Data analysis followed a narrative approach with not only a focus on what was told, but also on how this was constructed in interaction with the interviewer, the cultural resources that were drawn on and inconsistencies or alternatives that were presented. This narrative focus was used to explore how professionals made sense of their experiences with PA counselling within the wider sociocultural context.

**Results:**

While the professionals view PA as a foundation of type 2 diabetes treatment, they experience it to be a tricky subject. Two main areas of tension were identified: (1) the understanding of patient behaviour; and (2) professionals’ views on responsibilities, both on their responsibilities as professionals and their notions on who is responsible for behaviour change.

**Conclusions:**

Health care professionals providing PA counselling to people with type 2 diabetes have to navigate between possibilities within the diabetes care framework, options for an embedding of PA in the patient’s lifeworld, and the professionals’ opinions on and experiences with PA and healthy living from their own lifeworld. This makes PA a complex topic of care.

**Electronic supplementary material:**

The online version of this article (10.1186/s12913-018-3102-1) contains supplementary material, which is available to authorized users.

## Background

Physical activity (PA) is considered a ‘cornerstone’ in type 2 diabetes care, along with diet and medication [1]: one of the starting points for treatment is to ‘stimulate the patient to be sufficiently physically active and to work on the improvement of one’s fitness’ [2, 3]. This is based on numerous biomedical and epidemiological studies that point towards positive effects of regular PA on blood glucose control especially, as well as several other parameters important in type 2 diabetes treatment [4, 5].^1^

This emphasis on PA fits within a larger care framework on self-management, a key aspect of type 2 diabetes care [3, 6, 7]. Self-management is defined as the individual’s ability to gain control over an illness, amongst other things, by managing lifestyle changes and in conjunction with health care professionals and relevant others [8]. This, in turn, fits in current dominantly neoliberal government and health policies, in which the responsibility for well-being primarily lies with the individual and ‘consumer choice’ and ‘empowerment’ are considered central values [9].

In medical guidelines for diabetes care, professionals are instructed to advise, encourage and provide ongoing counselling on the topic of PA, as part of ‘diabetes education’ [3]. Overall, these strategies are directed towards increasing patient knowledge, for instance, on the benefits of PA and the possibilities to practise it. The assumption is that the patient will make the ‘right’ choice when offered enough information. This matches with a ‘logic of choice’ in which scientific evidence is considered neutral and clear, and people make independent, informed and rational decisions with a presupposed predictable outcome [10]. The task of professionals, then, is to provide their patients with this knowledge so that these can decide for themselves what might be the best treatment or way of life.

However, the same global guideline that stresses the importance of diabetes education also refers to its ‘patchy evidence’ on effectivity, especially doubts about its long-term effects, and acknowledges that promoting knowledge alone is not enough [3]. In fact, many studies point towards the difficulty of behavioural change. A study among almost 5000 women demonstrated that a diagnosis with diabetes or another chronic disease did not impact on their level of PA significantly [11]. Related to type 2 diabetes, this is even more complex, as physical inactivity and obesity are considered important causes [12]. This means that professionals are instructed to stimulate people who generally are not inclined to be active.

However, a complex combination of other factors are found to be important in the causation of type 2 diabetes, like genetic susceptibility, diet, in utero and early malnutrition and environmental factors [13]. This is further intertwined with intersecting social categories like socio-economic status and ethnicity. In high-income countries, type 2 diabetes is more prevalent among people with a lower socio-economic status, measured by education, occupation or income [14], and people with a specific migrant background, like people from Turkish, Moroccan or Surinamese descent in the Netherlands [15, 16]. In general, these people have more problems related to health and daily living conditions, like housing and work, than others [17]. These problems are also considered to preclude much attention to healthy living [18].

PA is a practice embedded in daily life. Therefore, a focus on PA counselling provides an example in which biomedical guidelines and daily life have to be brought together. This might produce certain frictions. A meta-analysis of qualitative studies about patients’ and professionals’ views on type 2 diabetes medication adherence, for instance, showed that professionals generally limit their focus to clinical issues, while patients mention problems from a much larger perspective including the personal, social and practical challenges of living with diabetes [19].

Moreover, PA is also something that professionals have personal experiences with, whether positive or not, and whether recent or long ago. These experiences might influence their tendency to address the subject at all, for instance, because of a personal affinity [20]. Furthermore, professionals probably also have personal opinions on the importance of PA, possibly influenced by a larger social discourse emphasizing that ‘sport is good for health’ [21]. Hence, the daily life of the professional might – implicitly or explicitly – also be present in the consultation room, or, in contrast, consciously left out [22].

Finally, professionals work within an established framework of care with a certain protocol and amount of time. In a systematic review, a lack of time was identified as the most common barrier for professionals to provide sufficient PA counselling in clinical practice, followed by a lack of knowledge or training and a lack of reimbursement [20]. Related to the care framework, Dutch nurses specializing in diabetes are ‘trained to organize their care efficiently’ [23]. They are considered able to deliver effective and efficient care for people with type 2 diabetes, with ‘effective’ referring to the improvement of certain measurable parameters like HbA1c [24]. These studies fit within a larger social and political discussion about increasing health care costs, considered relevant to diabetes since its prevalence is expected to increase in the next decades [25]. In this sense, PA might be considered a cheap ‘medicine’.

Notwithstanding these guidelines and all kinds of possibilities, difficulties and limitations, health care professionals have to act; they have to deliver PA counselling or care to their patients. Most studies among diabetes professionals aim to improve patient outcomes or adherence; for example, by identifying barriers and enablers to PA counselling [20] or factors influencing PA promoting practices [26]. They do not examine the experiences of professionals. Insight into these, however, might provide us with valuable information about frictions and concordances in diabetes care from a professional perspective. This might lead to a better understanding of what ‘good care’ implies and what is needed to offer this [10]. In the long term, these understandings might add to the well-being of both professionals and their patients.

Type 2 diabetes care in the Netherlands is found to be of a very high quality [27]. There is a National Care Standard for type 2 diabetes [28], which is strictly followed by primary care providers [27], and a specific guideline on type 2 diabetes for general practitioners and practice nurses [2]. These both emphasize the importance of PA as a topic of care. Since self-management is found to be an explicit focus in Dutch diabetes care [29], it is expected that PA is a standard topic of care. This makes the Netherlands an interesting case for this study. Therefore, the aim of this article is to give in-depth insight into experiences of Dutch health care professionals with the delivery of physical activity counselling to people with type 2 diabetes.

## Methods

### Design

To gain profound insight into the experiences of health care professionals, this study has a qualitative and narrative design. It is based on in-depth interviews with professionals providing care to people with type 2 diabetes. These interviews were considered stories in which the professionals selected, connected and evaluated experiences they considered meaningful in the context of this study [30]. This narrative focus is useful to explore how professionals make sense of their experiences with PA counselling within the wider sociocultural context [30]. This is of importance because this context offers possibilities, difficulties and limitations related to PA and PA as a topic of care.

### Pilot study

In 2012, a pilot study was conducted by another researcher [31]. This study was co-supervised by the author. Professionals were recruited through the networks of the researchers and the Internet. In total, 22 were approached and 11 agreed to participate. Four of them were practice nurses in a health centre or diabetes nurses in a hospital and the others offered actual PA care, like physiotherapists. The main interview topics concerned personal and professional experiences with sport and physical activity [see Additional file 1]. The interview took place at the respondent’s workplace, and was recorded and transcribed verbatim. The transcript was sent back to the respondent as a member check and reactions were added. This pilot served as input for a research proposal to extend the study on this topic and to test the interview format. Based on this, the format was adjusted; however, the background of the study and the general interview topics remained the same. Therefore, the transcripts of the pilot study interviews were added to the main study during data analysis.

### Data collection of the main study

The main study was conducted in 2014-2015 by the author. She has ten years of experience in qualitative research in the fields of sport and health. Sixteen professionals were approached by email or telephone. They were recruited through several (diabetes) organisations (*n* = 9) or the Internet (*n* = 7). Sampling was purposive in nature. Some professionals were asked because of specific experiences; for instance, because they offered a discussion group for people with diabetes or organised a weekly walking or exercise group. The aim was to include a wide range of professions within diabetes care. However, there was an emphasis on practice nurses and diabetes nurses – since they generally provide most of the care to people with diabetes in the Netherlands^2^ – as well as on those who offer actual PA care, like physiotherapists. Furthermore, the aim was to include at least eight professionals providing PA care and eight professionals offering PA counselling. Professionals were not asked about their own PA levels or experiences beforehand. Thirteen professionals agreed to participate; the others did not respond.

At the beginning of the interview, the respondent was encouraged to tell about his or her experiences and told not to expect many questions [32]. Using cards representing different domains – such as work, home, leisure time, experiences as a patient, and sport and PA – the respondent was asked about different roles in life. This was meant to stimulate the professional to shift ‘narrative positions’ throughout the interview and think about experiences with PA and sport from different perspectives [33]. The respondent was asked to tell about their role(s) at work and experiences with offering PA counselling, followed by PA experiences outside the work domain and a reflection on intertwinements between these work-related and other experiences [see Additional file 1].

Dependent on the available time of the respondent, the interviews lasted between 30 min and two hours, with an average of an hour. Two interviews took place at the respondents’ homes, the remainder at their work places. Afterwards, the interviewer noted down experiences with and feelings about the encounter in a reflexive account and transcribed the interview verbatim.

### Data analysis

Data analysis started after the first interview. After listening to the audio and re-reading the transcript, the author wrote the story as told by the professional, staying close to the words of the respondent and the order of the conversation. This story also included questions asked, to show how it developed in interaction. Furthermore, it included a preliminary analysis. This account was discussed with one or two other researchers involved in the larger project and sent back to the respondent as a member check. Three of the respondents did not respond after a reminder. Since a reaction was voluntary and they had not withdrawn from the study, their transcripts and stories were included in the data analysis. The reactions of the others differed from ‘difficult to read my own spoken language’ to ‘very nice account’. Besides some minor suggestions, all agreed on the account as presented.

From the first interview onwards, specific areas of tension or dilemmas related to the professionals’ experiences with PA counselling seemed to arise. In subsequent interviews, these were confirmed, enriched and supplemented. After 13 interviews, data saturation was observed. Although new stories were told during the last interviews, the main difficulties and areas of tension related to PA counselling or PA care were the same.

The overall analysis consisted of an iterative process – moving back and forth between stories and transcripts and between individual and general level – aligning with a narrative approach: with not only a focus on what was told, but also on how this was constructed in interaction with the interviewer, the cultural resources that were drawn on and inconsistencies or alternatives that were presented [30]. Data were coded using MaxQDA, version 12.0. This was mostly inductive, with the aspects of the narrative approach in mind. The stories were first coded, followed by the transcripts to check if all important information was present. At this stage, the transcripts of the pilot interviews [31] were added. These further confirmed the findings. Within this diverse group of professionals, data saturation was felt to be reached. Nonetheless, for a specific comparison between different groups of professionals – which was not an explicit aim of this study – more professionals within the groups should be interviewed.

In the end, two main areas of tension were identified. These were: (1) the understanding of patient behaviour; and (2) professionals’ views on responsibilities, both on their responsibilities as professionals and their notions on who is responsible for behaviour change. To check the analysis and interpretations, earlier versions of this article were discussed with several other researchers from different disciplines, like sport sociology, care ethics, anthropology and health psychology. To illustrate the findings, and especially reveal the complexities the professionals testified about, some of them are quoted at length in the results section.

### Ethical considerations

This study is part of a larger project, Sport in Times of Illness, which was approved by the Medical Research Ethics Committee of the VU University medical centre. The respondents received a letter with information about the aims of the project, the interview, and details concerning anonymity, confidentiality, audio recording and the option to withdraw at any time. They signed informed consent. All transcripts and stories were anonymized and participants were assigned pseudonyms.

### Participants

The total sample – pilot and main study – comprised 24 Dutch professionals providing care to people with type 2 diabetes: eight physiotherapists, five practice nurses working in a health care centre, three diabetes nurses working in a hospital, two general practitioners (GPs), an internist, a nurse specialist, a dietician working in a diabetes centre, an exercise coach, an exercise expert working in an obesity clinic and a health specialist working in a health centre. Seven of them were male, their mean age was 44 years (range 25-64) and they had 15 years of work experience on average (range 1-40).

## Results

Regarding the place of PA in diabetes care, most of the professionals noted it was something they almost always mentioned to their patients or asked them about, as ‘the foundation of treatment’:

*For all topics [in diabetes care] the same applies: there are always more or less the same advices, namely, do more PA and take care of your diet. But PA, that’s a tricky subject. It is not something people are easily inclined to. It’s difficult to change, always. (Gemma, nurse specialist)*

This quotation is illustrative of the findings in several ways. It underlines the importance of PA in diabetes care according to the professionals, but the use of ‘tricky’ refers to difficulties and tensions that accompany PA counselling, at least from the perspective of the professionals. It seems to be something that challenges them. This is exemplified by the two main areas of tension found in the professionals’ stories: the first is the understanding of patient behaviour and related difficulties that the professionals encounter; the second is the professionals’ views on responsibilities, both for behaviour change and as professionals. These two areas overlap at times and are further explained in this section.

### Understanding patient behaviour

The stories displayed a lot of references to understanding patient behaviour, varying from some understanding to none at all. The analysis revealed that these references mainly reflected differences the professionals had experienced between themselves and their patients, both implicitly and explicitly mentioned. These were differences in experiences with and meanings assigned to sport and PA, opinions on healthy living and taking personal responsibility for health, and social positions. Understanding was influenced by both personal and professional experiences that made it more or less easy for the professionals to identify with patient behaviour.

For instance, some professionals had personal experiences that increased recognition of their patients’ difficulties to become more active. Three professionals could not practice their favourite sport anymore because of an injury or illness and discovered that they ‘hated’ to go to the gym. Two others had other priorities at the moment – a mother being ill, a young child at home – and noted that they would like to be more active, but were not able to realize it. A GP explained that she started running and found it very difficult, especially because of the muscular pain. This made her realize that her patients needed someone guiding them, for example, a physiotherapist, to tell them, ‘This is normal… You need a week to get used to this’ – an addition that may also reflect a distance between her, having a lot of experiences with sport, PA and probably aching muscles, and her patients, mostly without these.

Based on their work experiences, several professionals came to understand that the goal of the medical guideline – being moderately active for 30 min a day [2] – was ‘unrealistic’ for many of their patients. They emphasized that ‘small steps are also steps’ and discovered that for most patients, especially those who were not used to being active, it was important to ‘think’ in small steps. These were preferably steps that could be integrated in daily life, like cycling to the store instead of taking the car. Some professionals mentioned that they often tried to slow patients down, because otherwise ‘they’ve all kinds of excuses why they didn’t succeed the next time’. Nicole (dietician) emphasized the importance of small, realistic goals, while she also noted that many of her patients did not know they should be active for an hour a day to lose weight. A goal quite difficult to attain, she added.

Some other professionals, especially the physiotherapists, did keep the norm of 30 min a day in mind: they provided trainings for three months twice a week and encouraged patients to do something else at home in order to meet the standard. However, some of them experienced this to be quite difficult for patients without positive PA experiences: ‘It is a utopian dream to think you have it fixed in 12 weeks.’ This shows a tension between guideline and practice that leads to other understandings of what to expect from patients.

Another tension was related to differences in views on healthy living. Some professionals held quite strong views about their patients, especially the physiotherapists – who seemed to experience the greatest differences between their own and their patients’ behaviour and to disqualify the lifestyle of their patients most strongly:

*Every now and then I have conflicts with those people, because they have a totally different approach on how to keep your body healthy. And that’s difficult. (…) I find health a privilege. (…) I think that if you get a good package from home, you need to treat it decently. So that means: don’t stuff yourself with that much food and burn enough calories. (…) I find it very hard that people have so much difficulties with this. (John, physiotherapist)*

*The average diabetic is not willing to be active. Now, I put it very black and white; that’s not nice to say. The average diabetic has overweight[ness], comes from a lower SES group. In general. He has no money to do sport, or isn’t willing to pay for it. Because they have money for a big flat screen. But yeah, they give low priority [to PA]. You have to see it this way, or I see it this way. (…) It irritates me. Of course, you have problems with your ankles and knees, you are 30 kilos overweight. (…) I think: ‘That’s just not necessary, that you became that fat. Where did it go wrong?’ I experience this from my own perception, my own situation at home with two incomes, nice house. A totally different perception than from someone sitting at home. And I cannot look behind the front door, that’s the problem. (…) Everybody can be active (…) just walk or cycle. (…) Priority, that’s what it is all about. (Marcel, physiotherapist)*

Especially, Marcel seemed to have difficulties in understanding his patients, while also being conscious about his different social and economic position. In the story of Krista (physiotherapist), the tension between understanding patients who are not willing to do something for their health on the one hand and realizing the difficulties of behaviour change on the other seemed to be the most palpable:

*Actually, given the fact that I’m working in a deprived area [where a lot of people with diabetes live], I find it surprising how difficult it is to get a group of people together [for my training programme]. And then, it’s often difficult to let people be here every time. Easy excuses to not be there again, you know. Not legitimate reasons, in your eyes, but yeah, what’s legitimate? For someone else, well, ‘the neighbour is here to drink coffee [so I cannot be there]’. (…) So how important is PA for you? To keep on going, that’s quite difficult [for me], to keep the motivation up. (…) And I let them pay 25 euros, as a sort of promise [to keep coming]. But there is a lot of moaning – that’s a big word, but they find it a burden. And then I think: ‘Well, hello, you can do trainings for three months for this 25 euro[s].’ (…) I don’t totally understand this. Do you really want to change something about your health or not? And isn’t this worth it? While you might make other decisions you easily pay 25 euro[s] for. (…) You flog a dead horse sometimes, to put it disrespectful[ly], and you don’t feel like it. I want to give some input, but you have to want to be here yourself. (…)*

*But it is quite difficult to do exercises at home consequently. I find that hard myself, too. Sometimes I think: ‘Now, I’m going to do stomach exercises for a while.’ Well, a week is over before you know and you think: ‘Ooh! I’d do those exercises! Wait, it is like this for that patient as well…’ (…) So I get that for someone with already a difficult body, to realize this, that’s far from easy, when you’re 140 kilos, to start being active. We’d carry three backpacks, or maybe even four. ‘Go and train on that cross trainer.’ Well, that’s hard (…) And that stupid homeopathic pill I needed to take twice a day, how many times did I forget it? That really was an eye opener for me. What are you expecting from everybody? So, you can say, ‘Do your exercises daily or go and be some more active every day’, but between good intentions to do so – because I really wanted to take that pill – and doing it, that’s not so easy. It’s quite difficult. Man is such a creature of habit.*

She seemed to be disappointed and frustrated by the lack of enthusiasm for the programme she offered. However, some of her own experiences made her gain an understanding of her patients’ difficulties as she could better identify with these. This quotation clearly illustrates certain contradictory feelings related to the understanding of patient behaviour and the difficulties professionals experience.

Most of the professionals balanced a bit between understanding and at the same time not really understanding the behaviour of their patients. Several emphasized that ‘PA is something extra, it comes on top of it all’, as people with type 2 diabetes also have to take care of medication, diet and sometimes try to quit smoking. Three others mentioned that most of their patients do not feel ill or are not bothered by their diabetes in daily life, which makes it ‘hard to constantly keep the focus on diabetes’ or see the necessity of continuing PA. Many noted the importance of positive childhood experiences in sport and PA, something they almost all had themselves, but knew many of their patients did not. Financial barriers were mentioned a lot, and a GP added that sport can be quite expensive, especially when you are not used to paying for it. Some professionals referred to socio-economic or cultural differences between them and many of their patients, like a lower socio-economic status, living in a deprived neighbourhood or having a non-Western background, especially for women. These patients were thought to have more difficulties in understanding the messages, to set certain ‘priorities’ and to implement PA in daily life. Although this seemed to result in a sort of understanding or acceptance on the one hand, it also made it difficult for the professionals to really understand the difficulties on the other hand, because they were not familiar with these circumstances.

These differences were also visible in the way the interviewer was sometimes included in the story of the professional: ‘of course, we easily talk about PA’, Paul (physiotherapist) said to the interviewer, without knowing anything about her PA story. This illustrated a presumed difference between him and the interviewer on the one hand and his patients on the other hand. Sarah (practice nurse) formulated it this way:

*You just have two categories of people: those who sit behind the wheel, who want to have control over their life, have their own responsibility and take it, and there are people who sit in the back of the bus and let themselves be driven, who let it happen. And if those people don’t undergo a change [in attitude], they have a long way to go before they get in at the front of the bus, sit there. Then you have such a different way to go before you even talk about PA.*

Sarah was very active herself, in contrast to a share of her patients. Although she was conscious of this difference and reflected on it throughout her story, at times she had difficulties in understanding patients who seemed to have another view on taking responsibilities for their health. This is where the tensions between the themes of understanding and responsibilities overlap.

### Responsibilities for behaviour change and professional responsibilities

Within the theme of responsibilities, several questions or tensions seemed to arise: who is responsible for behaviour and behaviour change? What are professional responsibilities and what are not? How far do these responsibilities go? Several professionals talked about changes in their profession over the years related to this theme; for instance, in the way of communication with a growing emphasis on ‘motivational interviewing’ – instead of telling patients what to do, they have learned to ‘lean backwards’ and let patients tell them what they want to do. Alice (diabetes nurse) experienced a transition from being a professional who knows what to do towards being a kind of counsellor now. She also saw the attention to behaviour change in diabetes care increase a lot. The three physiotherapists with more than 25 years of work experience noticed a comparable transition – from giving massages and ‘fixing’ patients to letting them be active during therapy and searching for a solution, namely becoming more active in daily life, together.

Overall, tensions related to responsibilities developed mainly from doubts or ideas about when it is time ‘to let someone go’. As Emma (just started working as a physiotherapist) put it:

*That’s frustrating sometimes, that the GP, the practice nurse, the physiotherapist… Everybody puts so much energy, time and enthusiasm into it. And if someone really doesn’t want to, do we have to keep on trying? Or do we need to say, ‘It ends here’? Luckily, I didn’t experience [that someone really didn’t want to]. (…) I didn’t see them, but I believe they exist.*

This quotation also illustrates a professional image of specific patients: those who do not want to be active. In the end, the professionals considered it to be the responsibility of the patient – ‘if people don’t want to, that’s fine, then it stops’. However, they seemed to differ regarding the possibilities they could offer as a professional. For Nicole (dietician), it was clear that her patients needed help, otherwise they would not come and see her, although the only things she felt she could offer were advice and discussions about the topic. After 40 years of work experience, Willem (internist) had ‘little illusions’ left:

*Type 2 diabetes is a consequence of our lifestyle. So here you see the development of a chronic illness as a consequence of your own behaviour. (…) Getting people to be active is a public health care responsibility. My responsibility is to offer these people, when they have developed diabetes, their care as good as possible (…), not to change their behaviour. Even if you do your absolute best, it won’t happen. That doesn’t mean you don’t have to try, especially with younger people and people you can have a good conversation with, it sometimes happens. But the alteration of people is strongly dependent on the environment they want to and can live in, and that’s something I cannot change from my health care position. (…)*

*I quit saying, ‘It’s nice to do some exercise’. Sometimes, I propose: ‘How about cheating? Let me send you to a physiotherapist, so you have to do it for a while.’ But then [when the insurance fee ends after three months] people tell me, ‘I quit, because I didn’t get it paid anymore’. Well, yeah, you can walk outside, guys. That’s what you get, especially people with type 2 diabetes, nine out of ten revert to the same habits. So, I’m pessimistic about it. But in this case, it’s realistic. (…) I do my best for the people I see, but, in the end, I won’t create a solution with my advices.*

He mentioned the need for professionals to see some results of their efforts in order not to lose motivation, a ‘what’s in it for me?’ for professionals. Although he hinted at the rare possibility of results, he seemed to have let go of feeling responsible for the behaviour change of his patients, as he experienced it to be beyond his reach. He was the only professional who explicitly expressed this view. He did not consider behaviour change the responsibility of his patients alone, either, but also of public health care, although the ‘you can walk outside’ remark reveals an ambivalent attitude towards this.

As a limitation to professional responsibilities related to PA counselling, several professionals mentioned characteristics of the health care system, like lack of time, low frequency of appointments or a focus on protocols instead of people. Especially, Gemma (nurse specialist) considered ‘the system very difficult at the moment’, with an increasing emphasis on administration and high demands of insurance companies. Willem (internist) mentioned both time and the focus on protocols as difficult:

*I’ve only ten minutes and more to discuss. So that’s too little [time] to seriously talk about it. (…) My first question is: ‘How are you?’ And they give me their sugar levels. That’s not what I asked. (…) Our practice nurses are also drilled to focus on bringing those sugar levels down. That’s why we have the best diabetes care in the world, but now it’s time to look more at the person instead of its numbers.*

Elena (practice nurse) admitted that she needed the protocol as something to hold on, but at the same time found it difficult because ‘that whole list is not the list of the person [themselves]’. She found a way in between: after finishing the protocol, she asks people how they are.

The limited overview and possibilities of PA options to refer patients to were also mentioned several times. Professionals considered the possibilities as often temporal because of subsidies that ended, and therefore found it difficult, and also not necessarily their responsibility, to keep the overview up to date. A frequently mentioned subject was the temporariness of physiotherapy – because insurers cover three months of training – and the difficult task for patients to continue to be active afterwards. This was not so much mentioned by the physiotherapists themselves; most of them told about their patients being enthusiastic during trainings, but losing them out of sight afterwards. However, the other professionals – those who referred patients to the physiotherapist and saw them back afterwards – experienced that only few people were able to continue to be active on their own.

A specific theme that emerged among the ‘consultation room professionals’ – those who did not offer PA trainings – was a tension between the wish to offer people something more than only advice or to consider this not a responsibility at all.

*If the step to start is too big, I cannot do anything else. I can hardly take them by the hand and go with them, can I? So, then it’s with them. I can give some advice, but they need to get going themselves. I’d prefer to say, ‘You have to do this and that’s it – easy’. But it doesn’t work this way and that will always be difficult. (Alice, diabetes nurse)*

*I’m a GP, so I’m not someone who actually goes along with people if they have to be active. A GP often gives advices, discusses resistance, doubts or constraints and gives explanations about the importance of being active. (Thomas, GP)*

At the moment of the interview, however, Thomas was involved in the organisation of a walking group. However, he tried to do this together with volunteers and social work, as he did not consider it a responsibility of him and his colleagues to keep such a group going. Elena (practice nurse) started a weekly walking group herself, because she wanted to offer her patients something concrete and accessible instead of ‘only telling them to be more active’. Marjolein (practice nurse) also started such a group. Both spent some of their spare time on the organisation. They were enthusiastic about their group, and felt it offered something important for some of their patients, both because of the physical activity and the social aspects. However, they also considered it quite a lot of effort to keep it going and find new people to join, and sometimes felt frustrated by this. Especially, the story of Marjolein seemed to represent different tensions related to the theme of professional responsibilities and the motivation to keep going as a professional:

*Actually, people prefer to be picked up at home, taken by the hand and properly brought back home again. Yeah, society changed; that’s not possible anymore. (…) So now I’m letting it go a little bit; it needs to be initiated by the patients as well. And sure, I can meet someone halfway, help them to get on board, but people who are not motivated to be active, that’s such a waste of time. Really, that’s not fun for both of us. (…) At the front door, you need to be able to assess if someone is motivated or not. And with those who are, you go on. And those you think are not, or people who think they won’t achieve anything, you have to let them go. That’s the tendency at the moment. (…) I’ve heard all [the] excuses by now. So that makes it very, very difficult to stay motivated [for me]. To get going every time. To keep on walking. (…)*

*Sometimes, it just doesn’t work. There are people who say, ‘I don’t feel like being active, I don’t see the benefits’. Then you talk about this several times to make the benefits clear. And if they still don’t want to, well, fine, then it ends. Then it’s not my responsibility anymore. I tell people sometimes: ‘It’s your chronic disease. You can see me as a kind of supermarket to get knowledge and information from, but you have to deal with this. (…) But don’t say that you didn’t know later on.’ Because this is something that is made understood good and proper to such an extent, that nobody can say that. So, they know, but it’s just what they want with it themselves. It doesn’t make me feel forced to… well, I always talk about it, but if they don’t want to… (…) I need to be sure they understand they can do something about their illness themselves by being more active and eating healthier. But if they know and do nothing about it, well, then it’s their own responsibility. (…) I mean, I won’t get paid any less or something.*

This ‘letting people go’ and assessing the motivation at the beginning, was something she learned over time: ‘Yeah, you also change in this process.’ Some other professionals also told about this:

*I learned it not to be my responsibility. In the beginning, you want to save people. (...) There is so much possible! You can do so much about it yourself! So, you are inclined to expect someone else to have your own norms as well, norms about health and taking responsibility yourself. (Sarah, practice nurse)*

This illustrated a shift in feelings of responsibilities over time, something Krista (physiotherapist) deemed necessary to ‘stay healthy as a health care professional’.

## Discussion

Physical activity is part of the medical guidelines on type 2 diabetes and as such needs to be integrated in the diabetes care framework with certain protocols and amounts of time per patient. However, it is also part of the patient’s lifeworld as a daily practice, either as something being practiced or not, and something someone wants or intends to do more or not. Health care professionals are positioned in between this medical world and the patient’s lifeworld [10]. Moreover, they also have experiences with PA and opinions about healthy living and related responsibilities from their own lifeworld. In the health care setting, professionals have to navigate between these different worlds. This makes PA a complex topic of care, as is illustrated by the results of this study among 24 professionals working in type 2 diabetes care in the Netherlands.

This study revealed that this complexity arises from a professional view on PA as a ‘foundation’ of diabetes care on the one hand and an experienced ‘trickiness’ of the subject on the other hand. It is shown in tensions concerning difficulties in understanding patient lifestyle behaviour, and the professionals’ – at times ambiguous – views on the responsibilities of patients and themselves. The professionals expressed rather ambivalent feelings about these themes, like the internist who had few illusions left regarding his possibilities to help patients change their PA behaviour, although he still stressed that one always needs to try. Or the practice nurse who started a walking group to extend her offer, although she found it hard to not lose motivation herself.

These tensions seem to increase when the professionals’ own behaviour and opinions differ more from those of their patients. Lifestyle practices, like sport and PA, as well as views on healthy living and responsibilities for health are related to social positions; in the Netherlands, as in other high-income countries, people from the lower social strata are generally less active and tend to attach less importance to healthy living and individual responsibility [34]. These factors are intertwined in an intricate way, and also present in the health care setting. Experiences with and benefits from health care closely link to social positions [17]. Tronto stated that we preferably take care of those close to us, because their needs are easier to assess [35]. Dutch professionals working in type 2 diabetes care – higher educated, and more often white people – differ from a share of their patients in background characteristics and living conditions, since type 2 diabetes is more prevalent among people with a lower socioeconomic status and a specific migrant background [14–16].

Although this study shows that professionals are aware of these differences and take them into account, the results also indicate that these differences make real understanding of the conditions patients live in and their PA behaviour difficult. This seems to make it complex for professionals to identify with patient choices and establish their precise needs. While many studies point towards a positive relationship between a professional’s personal interest in PA and the inclination to address the subject in the health care setting [20, 22, 36], this study demonstrates this might also make it more difficult to understand those patients who are not active.

Especially, the (male) physiotherapists seem to experience great differences between their own and their patients’ PA behaviour. Their personal interest in sport probably contributed to their choice of profession, while they particularly see those patients who have trouble being active. They offer PA care for 12 weeks because of insurer options and lose most patients out of sight afterwards; they offer an exercise ‘bubble’ in a treatment context, but do not see much of the ‘battle’ patients experience at home [37]. However, their sole focus on getting people more active and the realization that they cannot ‘fix it’ in this short amount of time might add to their frustration.

A quantitative study suggested that primary health professionals hold their patients, and especially those with type 2 diabetes, responsible for lifestyle change, but also experience this to be an unrealistic expectation [38]. The current qualitative study provides more insight into this dilemma. While professionals experienced an increased attention to behaviour change in type 2 diabetes care over the years, their main repertoire – giving advice, discussing the topic with patients, providing them with information, and referral to (or, for some, offering) temporary PA trainings – seems insufficient to meet the needs of a share of their patients.

This is something professionals are aware of and struggle with. Based on their experiences, they know that providing knowledge and advice alone is not enough, but they have limited alternative options. Some extend their offer, for example, by organizing a walking group, investing some of their own spare time, but they also experience difficulties and frustrations. This points towards the need of support and education for these professionals, to enable them to better meet the needs of their patients, especially those from lower socio-economic status groups or with migrant backgrounds. Support might be the availability of time or help from others, preferably those with expertise on the topic of PA and type 2 diabetes.

The professionals also seem aware of the importance of other factors, like early childhood experiences, financial barriers and socio-economic or cultural aspects. These are all found to be related to sport and PA behaviour [34, 39–41], and it seems necessary to increase the expertise of professionals on these topics in order to better meet the needs of these patients. For instance, by better integration of these topics in education or extra training, or by the inclusion of specific experts in the health care team, like exercise experts or people with experiential knowledge. The latter might also enable professionals to better understand their patients.

Recently, the Netherlands Scientific Council for Governmental Policy cautioned the government that the current emphasis on individual responsibility and ability to manage oneself seems to be too difficult for part of the population. They concluded that knowing what to do – for example, adopting a healthy lifestyle – does not equal knowing how to [42]. This was also suggested by a study among patients with chronic heart failure: patients did not need more information on what to do related to healthy living, but on how to do this in their daily life [43]. The current study shows that this is also applicable to health care professionals as it comes to the topic of PA in type 2 diabetes care: deeming PA counselling important is not the same as knowing how to offer this within the current diabetes care framework, with a certain available repertoire, other topics to be addressed, limited amounts of time and options to which to refer, and an emphasis on efficiently organised and effective care.

This makes professionals reflect on when it is time to ‘let a patient go’ and leave the responsibility to the patient. This study shows how professionals struggle with this, probably because it is contradictory to what they are trained to do [44]. According to Mol, what ‘good’ care exactly implies is not something universal, but needs ‘tinkering’: a process in which patient and professional together make a local fine-tuning of the guidelines by adjusting them to the daily life of the patient [10]. This requires not only listening to but also hearing patient stories, and trying and evaluating what works and what does not [10, 45, 46]. At times, ‘letting go’ of the topic of PA might also be good care, although professionals need to stay attentive to changing patient needs. Self-management practices are found to be ‘contextual, unlimited, and changeable’ [47]. Consequently, care practices need to have those characteristics as well. Moreover, as PA is not a ‘simple’ medicine to prescribe, this asks for sufficient equipment of, and time for, professionals.

### Recommendations

Three recommendations arise from this study. First, it points towards the importance of health care professionals being aware of their own norms, opinions and experiences that might impact their PA counselling practices and relationships with patients. A concrete suggestion for reflection might follow from the interview format used in this study as a means to think about or start a dialogue on care and counselling experiences as well as PA experiences in other life roles and possible intertwinements between these.

Second, walking groups organized by health care professionals seem to be an interesting extension of PA counselling. However, professionals might need extra education and support in order to enable them to better meet the needs of their patients. This is an interesting topic for further research.

Third, a risk of the current emphasis on PA, individual responsibility, and cost-efficient care combined with the tensions professionals experience in providing PA counselling is a demotivation of professionals. Given the expectations of an increasing prevalence of type 2 diabetes, this might become an even more urgent topic in the future [25]. Therefore, health care professionals need sufficient equipment and time to offer PA counselling and/or options to which to refer. Above all, they require recognition for the complexities they face in providing PA counselling – both for their own and their patients’ well-being.

### Strengths and limitations

This study has some limitations. First, the sample of respondents might, unintendedly, have a relatively positive attitude towards PA. Although participants were not selected based on their sport and PA behaviour, they received information about the study beforehand and the topic might have been a motivation to take part. However, only three professionals did not reply to a request to take part in the study: an internist, a physiotherapist, and a practice nurse with a walking group for patients. Furthermore, although other studies showed a positive relationship between a personal interest in PA and experiences with PA counselling [21, 23, 36], this study revealed insight into specific frictions related to personal PA experiences of professionals.

Second, the sample of 24 respondents is not representative of all professionals working in type 2 diabetes care in the Netherlands. Although saturation was felt to be reached during data analysis, the emphasis was on the experiences of practice nurses, diabetes nurses and physiotherapists. These are the professionals mostly involved in providing PA counselling or care. Therefore, in order to better represent the experiences of other professionals, probably more interviews are needed. This would also offer the option of comparison between professionals.

Third, in qualitative studies, the researcher is part of the process. This requires a reflexive awareness from the researcher. Therefore, several measures were taken to develop this: decisions regarding data collection and analysis were noted down as well as experiences with each interview; the individual stories of the respondents and earlier versions of this article were discussed with other researchers; respondents were given the opportunity to react on their accounts; and the findings were illustrated by long quotes from the respondents and related to the wider social context in which they were shaped. Furthermore, the personal and social characteristics of the author might have influenced the processes of data collection and analysis. She has a relatively privileged social position based on educational level, socio-economic status and ethnicity, like the respondents of this study and the interviewer of the pilot study. For instance, this might have made it easier in the interview setting to connect with the respondents. However, due to physical discomfort, she no longer participates in sport. This might have influenced her interest in this study in the first place.

Fourth, the professionals who participated in this study work in a specific diabetes care framework, namely in the Netherlands. This raises questions about the transferability of the findings to other contexts. Although this study is intended to offer in-depth insights instead of generalizable findings, it concerns themes that are currently emphasized in many countries, like self-management, individual responsibility for health, a search for an efficient and effective health care system and a tendency to consider PA as a means to become healthy. Therefore, the insights offered might also be of interest for health care professionals and researchers in other countries.

A considerable strength of the current study is the interview format, which invited the professionals to reflect on experiences with PA from different roles, both in their professional and personal lives. This made them reflect on the topic from different perspectives and evaluate their experiences from a broader point of view.

## Conclusions

This study provides in-depth insights into difficulties that Dutch health care professionals experience with the topic of physical activity in type 2 diabetes care. These insights reveal that PA can be experienced as a ‘tricky’ subject of care, because of two main areas of tension. The first encompasses difficulties of professionals in understanding the PA behaviour of their patients. These mainly seem to reflect differences that professionals experience between themselves and a share of their patients, especially in actual PA behaviour, opinions on healthy living and related social positions. These differences might cause certain frictions in the health care setting. The second is related to questions about and struggles with professional responsibilities, especially regarding doubts and ideas about when it is time to let go of the topic and leave the responsibility to the patient. These two areas of tension arise in a field in which professionals have to navigate between possibilities that the diabetes care framework offers, options for the embedding of PA in the patient’s lifeworld, and the professionals’ opinions on and experiences with PA and healthy living from their own lifeworld. This makes PA a complex topic of care.

## Additional file

Additional file 1: Interview questions. Topic lists used in the pilot study and the main study. (PDF 61 kb)

## Abbreviations

GP: General practitioner
PA: Physical activity

## Footnotes

1 These and other studies report about physical activity, exercise and/or fitness. For the purpose of this article, a distinction between these is not necessary. For reasons of readability, the term ‘physical activity’ is used here.

2 In the Netherlands, people with type 2 diabetes are treated in primary or secondary health care settings, mostly dependent on the gravity of their diabetes and type of medication. In primary care, GPs have the final responsibility, but generally most care is handed over to practice nurses.
