# Supplementary material for: ‘Physical activity, that’s a tricky subject.’ Experiences of health care professionals with physical activity in type 2 diabetes care
Source: BMC Health Serv Res. 2018 Apr 23;18:297. doi: 10.1186/s12913-018-3102-1 (PMC5914058; doi:10.1186/s12913-018-3102-1)
Supplement: Supplementary file 1 — Interview questions. Topic lists used in the pilot study and the main study. (PDF 61 kb) [file 12913_2018_3102_MOESM1_ESM.pdf]

Physical activity, that's a tricky subject.' Experiences of health care professionals with physical activity in type 2 diabetes care

Additional file 1. Interview questions

1a. Topic list of the pilot study

Introduction:

- Introduce yourself and explain about the aims of the study: to gain insight in meanings people with type 2 diabetes attach to sport and physical activity and related experiences of healthcare professionals.
- Aim of the interview: to gain insight into the experiences of healthcare professionals with SPA, both in their professional and personal life.
- Explain about the audio: confidential, it is used to analyse the interview, all identifiable information (names, organisations, places) will be left out in transcripts/accounts. The participant will receive the transcript of the interview in order to check it. Ask permission to put the recorder on.
- Emphasize you want to hear the experiences of the professional.
- Any questions?

1. General questions
   a) Can you tell me something about yourself? (Age, education, profession)
   b) How do you relate to patients with type 2 diabetes in your work?
2. Topic: personal experiences with sport and physical activity
   a) What is the role of sport and/or physical activity in your life?
      - From childhood to present
      - What do you do, how often?

- What does it mean to you?

b) Does your outlook on sport and physical activity influence your work?

c) Does your work influence your outlook on sport and physical activity?

3. Topic: professional experiences with sport and physical activity

a) What is your outlook on the importance of sport and physical activity in relation to type 2 diabetes?

b) How do you communicate this to your patients?

c) How do your patients act on your advices and counselling?

d) What are your experiences with the ways diabetes influences sport and physical activity behaviour?

e) What are your experiences with the ways sport and physical activity behaviour influences diabetes?

f) Do you experience differences between patients (more or less sporty) and how they deal with their illness?

1b. Topic list of the main study

Introduction:

- Introduction (social scientist, not a (bio)medical scientist) and explain about the aims of the study: to gain insight in meanings people with type 2 diabetes attach to sport and physical activity and related experiences with healthcare, both of people themselves and healthcare professionals.
- Aim of the interview: to gain insight into the experiences of healthcare professionals with SPA, both in their professional and personal life.
- Explain about the audio: confidential, all identifiable information (names, organisations, places) will be removed in transcripts/accounts, all information (audio, transcript, other

accounts) is stored at a save network. The participant will receive an account of the interview as a check. Ask permission to put the recorder on.

- Emphasize you want to hear the experiences of the professional.
- Any questions?

1. Put the cards on the table: work – home – social life – spare time – physical activity and sport – patient – informal carer.
   a) Can you tell me who you are by explaining about the roles you have in life?
2. Ask about the professional role:
   a) What are your experiences with guiding patients in sport and/or physical activity?
   b) How do you advice patients about SPA?
   c) What goes well, what needs improvement?
   d) How are you educated to do this? Does this match your experiences? Are you sufficiently equipped (knowledge, materials, means, …)?
3. Ask about the other roles: what are your experiences with sport and/or physical activity [in the other role(s) mentioned at the beginning]?
4. Ask the professional to reflect on SPA in the professional role in relation to experiences from other roles (use of own experiences in contact with patients, similarities, tensions, dilemma's, etc.).
5. End of the conversation
   a) If not mentioned, ask about background: education, career, years of professional experience.
   b) Ask about experiences with the conversation and motives to take part in the study.
